# Endovascular Thrombectomy in a Young Female Patient With Atrial Septal Defect: A Case Report

**DOI:** 10.7759/cureus.83765

**Published:** 2025-05-08

**Authors:** Nobuhiko Arai, Kazunari Yachi, Ryutaro Ishihara, Takao Fukushima

**Affiliations:** 1 Neurosurgery, Takashimadaira Chuo General Hospital, Itabashiku, JPN

**Keywords:** acute ischemic stroke (ais), arteriovenous shunt, atrium septal defect, endovascular therapy (evt), migraine, oral contraceptives, young age stroke

## Abstract

Large vessel occlusion (LVO) in a young patient aged less than 30 years of age is greatly rare. When approaching stroke-like symptoms in younger generations, stroke mimics like epilepsy, chronic subdural hematoma, spinal cord nerve compression due to herniation of disk, or autoimmune neuropathy like Guillain-Barré syndrome or multiple sclerosis are definitely a great hindrance to rapid diagnosis. This condition is likely to increase the difficulty of accurate diagnosis and delay treatment for a stroke in combination with the rarity of strokes in young individuals. What is more, a delayed medical approach for autoimmune neuropathy, like massive steroid injection, could result in a worse prognosis. Oral contraceptives (OC) for migraine without aura are controversial for an increased risk of ischemic stroke. We experienced a highly instructive case of a young LVO patient for whom mechanical thrombectomy was performed. A 26-year-old female patient with a past medical history of migraine or depression had been transferred to the hospital complaining of left upper and lower limb paralysis. Magnetic resonance imaging showed right middle cerebral artery occlusion and cerebral infarction in the basal ganglia. Rapid endovascular thrombectomy was performed and resulted in thrombolysis in cerebral infarction (TICI) 3 by three passes. The patient was discharged without any neurological deficit on the 10^th^ day after admission. She was on OC pills in the last six months before the onset of symptoms. The value of D-dimer on admission was 3.9 mg/dl. We could not acknowledge the other stroke risk factors and suspected the paradoxical embolism as a mechanism of stroke, considering the patient's young age. Transesophageal echocardiography revealed a relatively wide atrial septal defect (ASD). The diagnosis of paradoxical embolism via ASD was made, and a direct oral anticoagulant was started. Closure for ASD was planned. A few months after the ASD closure, the anti-coagulant agent is planned to be ceased. Clinicians have to note that even young people with psychological disorders or migraine can suffer from LVO. More prudent prescription of OC pills for migraine patients is necessary.

## Introduction

Acute large vessel occlusion (LVO) is an important subtype of ischemic stroke that needs prompt neuroradiological intervention. In 2015, robust evidence to support the treatment was established [1]. The majority of LVO patients are of an older age [2]. LVO-related stroke is relatively infrequent among younger patients (<50 years) [2]. There are few reports in the literature about LVO patients aged 20 years or less [3], and as clinicians, we seldom see such a patient in real clinical settings. In addition, young patients are inclined to show stroke mimics [4]. Stroke mimics like chronic subdural hematoma, epilepsy, autoimmune neuropathy, and spinal nerve compression are illnesses that present symptoms like acute ischemic stroke (AIS). From a public health standpoint, the significance of young stroke is tremendous because it can lead to a higher medical total cost for a prolonged lifetime compared to elderly people, and the cost of absence from work is significant for working age groups [5]. Thus, prompt diagnosis leading to shorter onset to revascularization time, which can result in a favorable prognosis, is imperative. Up to now, though a number of studies elucidate effective methods or factors to discriminate AIS from stroke mimics [6], the emphasis on the possible existence of LVO cases in patients under 30 years of age seems to be insufficient. We present a case of a young female patient with LVO. From the experience of this case, we recommend considering the neurological status of young patients with migraine or psychological diseases. Furthermore, echocardiography should be performed for young AIS patients suffering from migraine, even without aura, if they are on OC pills.

## Case presentation

A 26-year-old woman was transferred to our hospital with left hemiparesis and motor aphasia, which occurred three hours before she arrival at the hospital. The patient was unable to move her left upper and lower limbs (manual muscle test 0/5) and could not express any meaningful words. 

The patient had suffered from migraine without aura every two weeks for the past five years. She had mild depression. Though she did not meet the criteria of major depression, a psychiatrist advised medications to relieve her depressive condition a few years ago (selective serotonin reuptake inhibitors (SSRIs)), which she took for a few years but discontinued. No remarkable family history or smoking/alcohol history was found. 

Although functional neurologic disorder (FND) or atypical migraine was suspected at first, she was promptly referred for a magnetic resonance imaging (MRI) test according to our hospital’s protocol. In our hospital, if a patient shows (i) hemiparesis and aphasia or (ii) hemiparesis and conjugate deviation, the “LVO” protocol is definitely applied regardless of age, gender, baseline modified Rankin Scale (mRS), or past medical history. LVO protocol is a way to expedite the management by performing an MRI test at first and cutting relatively less important tests like an electrocardiogram or chest x-ray. After the protocol administration, the door-to-revascularization time in our hospital was shortened by approximately 40 minutes compared to the period before the protocol was in place. 

**Figure 1:**
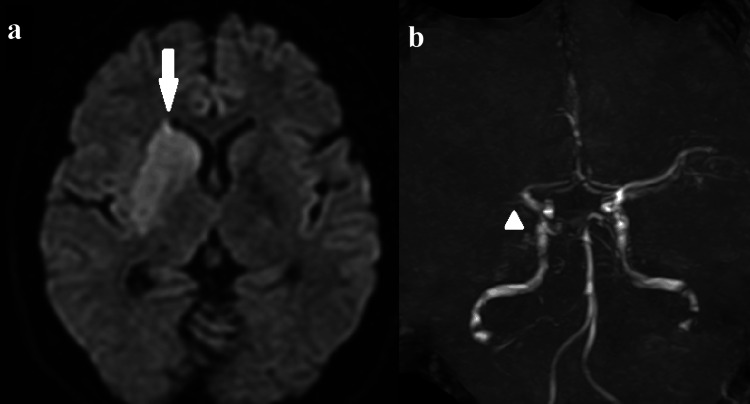
Initial MRI scan shows (a) high intensity area on the right basal ganglia and (b) the occlusion of right proximal middle cerebral artery.

**Figure 2:**
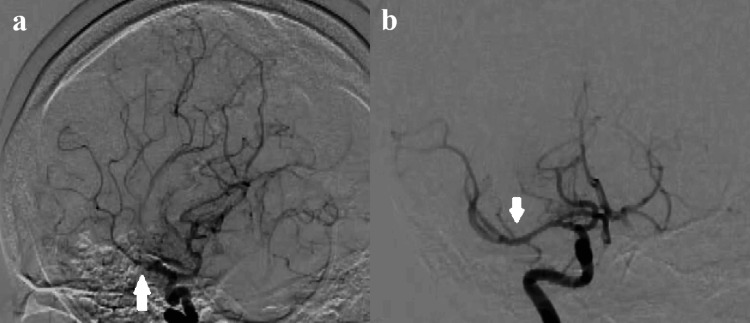
Angiography shows (a) the occlusion of right proximal middle cerebral artery (MCA) and (b) reveals the revascularization of occluded MCA.

**Figure 3:**
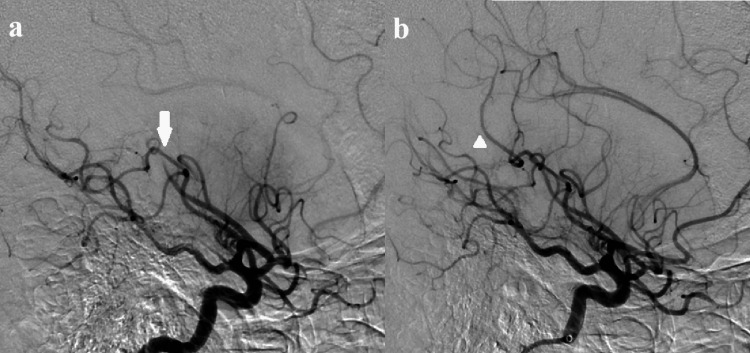
Final angiography images show (a) the occlusion of peripheral middle cerebral argery supplying eloquent area (motor cortex) and (b) the revascularization of occluded peripheral artery

The MRI image showed a high-intensity area in the right basal ganglia on diffusion-weighted images and the occlusion of the right proximal middle cerebral artery (MCA) on magnetic resonance angiography (MRA) (Figure 1). An endovascular thrombectomy was performed with local anesthesia soon after the MRI scan. After the one pass with a stent-retriever (Solitaire™ 6 mm; Medtronic plc, Dublin, Ireland) into an aspiration catheter (Catalyst® 6 Fr; Stryker Corporation, Kalamazoo, Michigan, United States), almost all MCA branches could be revascularized except for one eloquent peripheral branch to motor cortex (Figure 2). Finally, a small-sized stent-retriever (Trevo® 3 mm; Stryker Corporation) was used, and TICI 3 was achieved (Figure 3). It took 32 minutes to acquire the full revascularization from the femoral artery puncture. A few hours after the revascularization, the patient's hemiparesis steadily improved.

**Figure 4:**
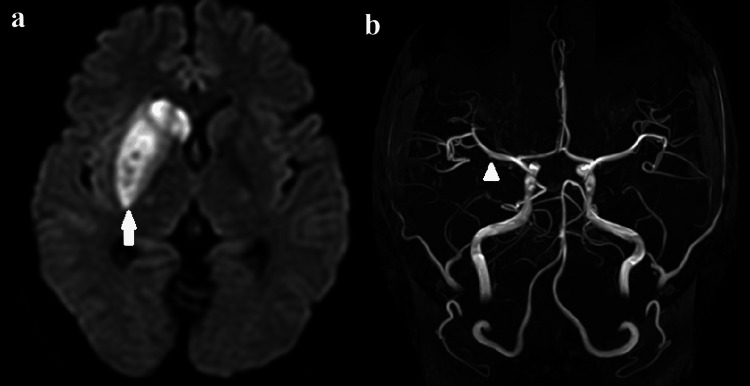
Postoperative MRI shows (a) intensified high-intensity area without any high area on the cortex after the endovascular sugery and (b) clear delineation of right middle cerebral artery

MRI performed two days after the intervention revealed no ischemic signs on the cortex (Figure 4). Three days after the operation, motor aphasia disappeared completely. Cognitive functional test was almost within normal range on hospital day 10, the day of discharge (Mini Mental State Examination: 28/30, National Institutes of Health Stroke Scale: 0/42, mRS: 1). 

**Figure 5:**
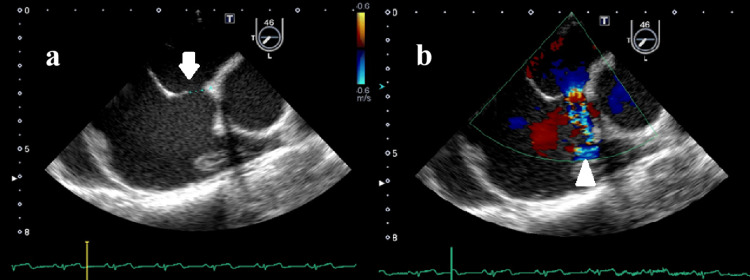
Transeshophageal echocardiography shows (a) atrium septal defect and (b) jet flow from left atrium to right atrium

Her intake of OC was found to be for six months by affirmative listening to her history in the ward. Considering young age stroke, various kinds of special blood tests such as protein C/S, anti-thrombin, anti-neutrophil cytoplasmic antibody, cyclic citrullinated peptide antibody, and homocysteine were performed and resulted in no abnormal values except for D-dimer (3.9 mg/dl) (Table 1). To exclude the possibility of paradoxical embolism, echocardiography and transesophageal echocardiography (TEE) were administered and showed no deep vein thrombus (DVT) but ASD (Figure 5). The size of ASD was 6.0x4.8 mm, with the ratio of Qp/Qs being 1.53. Even without detection of DVT, it was reasonable to suspect it as the potential embolus because it can sometimes completely disappear after its dispersion from the original site. Therefore, we made a diagnosis of paradoxical embolism via ASD due to DVT facilitated by OC. A direct oral coagulation agent was started, and OC was permanently ceased. Currently, endovascular therapy for closure of ASD is being planned. After the closure of ASD, the cessation of DOAC is scheduled.

**Table 1:** Result of blood tests CRP: C-reactive protein; HbA1c: glycated hemoglobin; LDL: low-density lipoprotein; BNP: B-type natriuretic peptide; ANCA: antineutrophil cytoplasmic antibodies; MPO: myeloperoxidase; AT III: antithrombin Ⅲ; PT: prothrombin time; INR: international normalised ratio; APTT: activated partial thromboplastin time; TSH: thyroid stimulating hormone

Parameter	Reference range	Patient value
White blood cell	3500～9000/μL	7500μL
Hemoglobin	11.4～14.6 g/dL	13.4 g/dL
Platelet	15.8-34.8×10⁴ μL	22.9×10⁴ μL
LDL	70～140 mg/dL	83 mg/dL
Triglyceride	30～149 mg/dL	84 mg/dL
BNP	≦18.4 pg/mL	71.7 pg/mL
MPO-ANCA	＜3.5 EU	≦0.5 EU
PR3-ANCA	＜2.0 EU	≦0.5 EU
Anti-cardiolipin antibody	<3.5 U/mL	1.2 U/mL
Protein S	63～135%	68%
Protein C	64～135%	96%
ATⅢ	80～130%	82%
Total homocysteine	5.3~15.2 nmol/mL	5.5 nmol/mL
D-dimer	＜1.0 μg/mL	3.9 μg/mL
PT-INR	0.9～1.1	0.97
APTT	30-45 seconds	27.3 seconds
Free-T3	2.13-4.07 pg/mL	2.95 pg/mL
Free-T4	0.95-1.74 ng/dL	1.15 ng/dL
TSH	0.34~3.88 μIU/mL	0.63 μIU/mL
C3	65-135 mg/dL	85.3 mg/dL
C4	13~25 mg/dL	15.2 mg/dL
Rheumatoid factor	0~15IU/ml	≦4.9/ml
HbA1c	4.6~6.2%	5.1%
CRP	≦0.14mg/dL	0.05mg/dL

## Discussion

We reported prompt diagnosis and successful endovascular thrombectomy for the young female patient. From this case, several vital lessons for clinicians can be found. First, it is necessary to exclude LVO if the patient shows focal neurological signs such as hemiparesis or aphasia, even though they are young in age. Second, the prime and important cause of juvenile stroke is right-to-left shunts like ASD, which is one of the most frequent adult congenital cardiac diseases. TEE is routinely necessary for young stroke cases. Finally, OC pills should be avoided for migraine with aura based on the WHO medical eligibility criteria [7]. Like in our case, patients with migraine without aura may suffer from a major stroke. Thus, a more prudent approach, like a less invasive AV shunt check or more aggressive periodical monitoring for thrombus, like D-dimer value, may be stipulated.

The prevalence rate of stroke in younger generations (<50 years) is estimated to be about 10-14% of all strokes [8], and there is a global increase in incidence of young stroke [9]. Generally, survivors of strokes experience disability, with a reported 60% of survivors being affected [10]. A meta-analysis revealed that around half of all young-stroke patients present with cognitive impairment and around a quarter with aphasia, whose ratios are not lower than those of older stroke patients [11]. Thus, certain appropriate measures for young stroke patients should be performed immediately. The effective strategy is assumed to be primary prevention and prompt diagnosis/treatment for stroke. In the current report, we discussed the latter. 

To get a prompt diagnosis and treatment, what can we do for younger strokes? A recent study showed young woman are at a disproportionately higher risk of ischemic strokes compared with their male counterparts [12]. A meta-analysis of 19 studies reported that there were 44% more women aged less than 35 years with ischemic strokes than men (incidence rate ratio, 1.44; 95%CI 1.18-1.76) [12]. Thus, nontraditional risk factors such as migraine, pregnancy, illicit drug use, and OCs must play a vital role in ischemic strokes of younger female patients. The patient in the current case had never smoked and had no traditional stroke risk factors such as hypertension, hyperlipidemia, or diabetes mellitus despite yearly medical checkups. When we see young female patients with stroke-like symptoms in clinical sites, nontraditional factors should be examined promptly. The examples of nontraditional risk factors are migraine, illicit drug use, oral contraceptives, and pregnancy [13]

In the meantime, there is a challenging problem in young stroke medicine: stroke mimics, which could complicate suspicion and result in hesitance and delayed diagnosis [4]. The patients whose past medical history includes stroke mimic diseases such as migraine, functional neurological disorders (FNDs), and seizure are likely to be triaged as a mild condition. The paper of the Angel-Act registry showed that the onset-to-puncture time was significantly longer in young patients, although there was no significant difference in door-to-puncture time and puncture-to-recanalization time between the younger patient group and the older patient group [2]. This fact reflected a prehospital delay. One possible explanation can be prehospital misdiagnosis by patients themselves, family or friends staying together with patients, or medical staff such as a primary home doctor or paramedic ambulance attendant. Several studies have reported that a few specific clinical symptoms can differentiate FNDs from acute ischemic stroke [14]. These factors may be helpful pre-image (MRI) screening scale to separate AIS from FNDs. From the precious lesson in our case, we have to recognize that LVO in seemingly healthy young female patients can be a possible existence if they have multiple nontraditional stroke risk factors such as migraine, smoking, OC pill use, AV shunts, and post-partum status.

Migraine is a common disease with a prevalence of 10-15% in the adult population. The first symptom is often in the teens, and the highest prevalence rate is in the age group of 20-50 years [15]. A recent meta-analysis showed migraine without aura does not carry a higher risk of stroke (relative risk (RR) 1.23, 95%CI 0.90-1.69). In the meantime, the RR of stroke in patients suffering from migraine with aura was 2.16 (95%CI 1.51-3.03) [16]. In addition, if migraine with aura exists with both smoking and OC use, the risk of stroke skyrockets sevenfold [17]. Currently, it is controversial whether stroke risk increases or not, if migraine patient without aura takes OC. The risk of venous thrombosis is reported to be highest within a 0.5-1 year period from the start of OC [18]. We suggest that for at least the first year, clinicians should perform a periodical monitoring for venous thrombosis when migraine patients start OC. In addition, AV shunt check may be necessary to prevent paradoxical embolism at the start of OC use, despite little evidence to support the cost and benefit of the test for them. It is reported that the rate of increase of congenital heart disease (especially ASD) in Asia (33.0% every five years) was significantly greater than in other regions [19]. Asian patients with migraine without aura taking OC pills in particular should take the test to exclude the cardiac AV shunt.

To the best of our knowledge, few reports exist in the literature about the relationship between ASD and migraine. The congenital shunt cardiac disease, similar to ASD, is patent foramen ovale (PFO), which can also be the cause of paradoxical embolism, like ASD. PFO is a frequent defect of the interatrial septum, with an incidence of 20-25% in the adult generations [20], with an incidence of 30-50% in those with migraine with aura in the meantime [15]. Thus, the relationship between PFO and migraine has been discussed. The reason behind PFO-induced migraine remains unclear. There are some hypotheses that hypoxaemia is a trigger of migraine attacks, or unmetabolized small neuro-active molecules facilitate abnormal cerebral blood flow [21]. Considering migraine hypothesis, ASD may be related to migraine attacks. ASD is the most frequent and increasing congenital cardiac disease among adults. ASD frequently does not lead to early diagnosis or referral because this disease is usually asymptomatic and has soft murmurs. This is why many ASD patients are diagnosed in adulthood. It is difficult to screen all migraine patients for the existence of a cardiac AV shunt. However, by limiting the subject to migraine, carrying high risks like OC use or smoking, primary screening for cardiac AV shunts like ASD or PFO seemed to be reasonable. However, there have been no studies to verify the cost-efficiency of primary screening to detect cardiac shunts for migraine patients with multiple risk factors related to ischemic stroke. Currently, there are three methods to detect PFO. Among them, transcranial Doppler is most useful and least invasive in the diagnosis of PFO-associated embolic stroke because it has a sensitivity of 91-100% and specificity of 78-100% [22]. Further investigation to confirm the cost-effectiveness of cardiac AV shunt check or assertive venous thrombosis check for patients with migraine without aura taking OC pills, should be performed in the near future.

## Conclusions

Prompt diagnosis of young stroke patients is important because rapid endovascular thrombectomy can be necessary even for these generations. Paradoxical embolism via cardiac AV shunts like ASD or PFO should be considered. Before prescribing OC pills for patients with migraine without aura, clinicians have to take a more prudent stance. AV shunt screening or more aggressive monitoring for venous thrombosis may be necessary despite the lack of robust evidence to support them. Therefore, future studies are needed to verify the cost-efficiency of these tests, considering the huge number of migraine patients.
